# CTpredX: Enhancing missense variant pathogenicity prediction in childhood cancer predisposition genes

**DOI:** 10.1016/j.gendis.2025.101661

**Published:** 2025-04-28

**Authors:** Ferdinando Bonfiglio, Vito Alessandro Lasorsa, Giampiero Pirozzi, Achille Iolascon, Mario Capasso

**Affiliations:** a Department of Molecular Medicine and Medical Biotechnology, University of Naples “Federico II”, Naples 80145, Italy; b CEINGE Biotecnologie Avanzate Franco Salvatore s.c.a r.l., Naples 80145, Italy

Effective clinical genome interpretation relies on accurately distinguishing between benign and pathogenic rare variants. Current machine learning-based variant prioritization tools are trained on genome-wide data and often overlook key parameters defining gene–disease relationships. Genes that cause a specific disease or a group of related diseases are likely involved in common biological processes. We hypothesize that these genes will share more features not captured by existing genome-wide tools. Disease-specific variant classifiers have been shown to outperform genome-wide tools when specifically applied to inherited cardiac diseases, inherited retinal diseases, or primary immunodeficiencies.^1^ However, no tool or predictor has been specifically designed for pathogenicity prediction of missense variants in childhood cancer predisposing genes (CCPGs).

To improve prediction accuracy for variants in childhood tumors, we developed a predictor for variants mapping onto a well-curated set of CCPGs. Trained on disease-specific data, the predictor CTpredX (called Childhood Tumor pathogenicity prediction with XgbTree) integrates multiple variant annotations, constraint scores, and pathogenicity predictors obtained from existing computational tools. It predicts the probability of missense variants being pathogenic (or benign) based on a machine learning algorithm, namely XgbTree.

To build the machine learning models (workflow in Fig. 1A), we restricted our analyses to a well-curated list of 333 childhood cancer-associated genes (Supplementary Methods; Table S1) and used three independent datasets: i) ClinVar variants with no conflicting clinical interpretation up to January 15, 2023, split into training and testing sets; ii) ClinVar data from January 15 to March 18, 2023, used for validation; and iii) missense variants from a cohort of 724 neuroblastoma cases,^2^ also used for validation.

**Figure 1:**
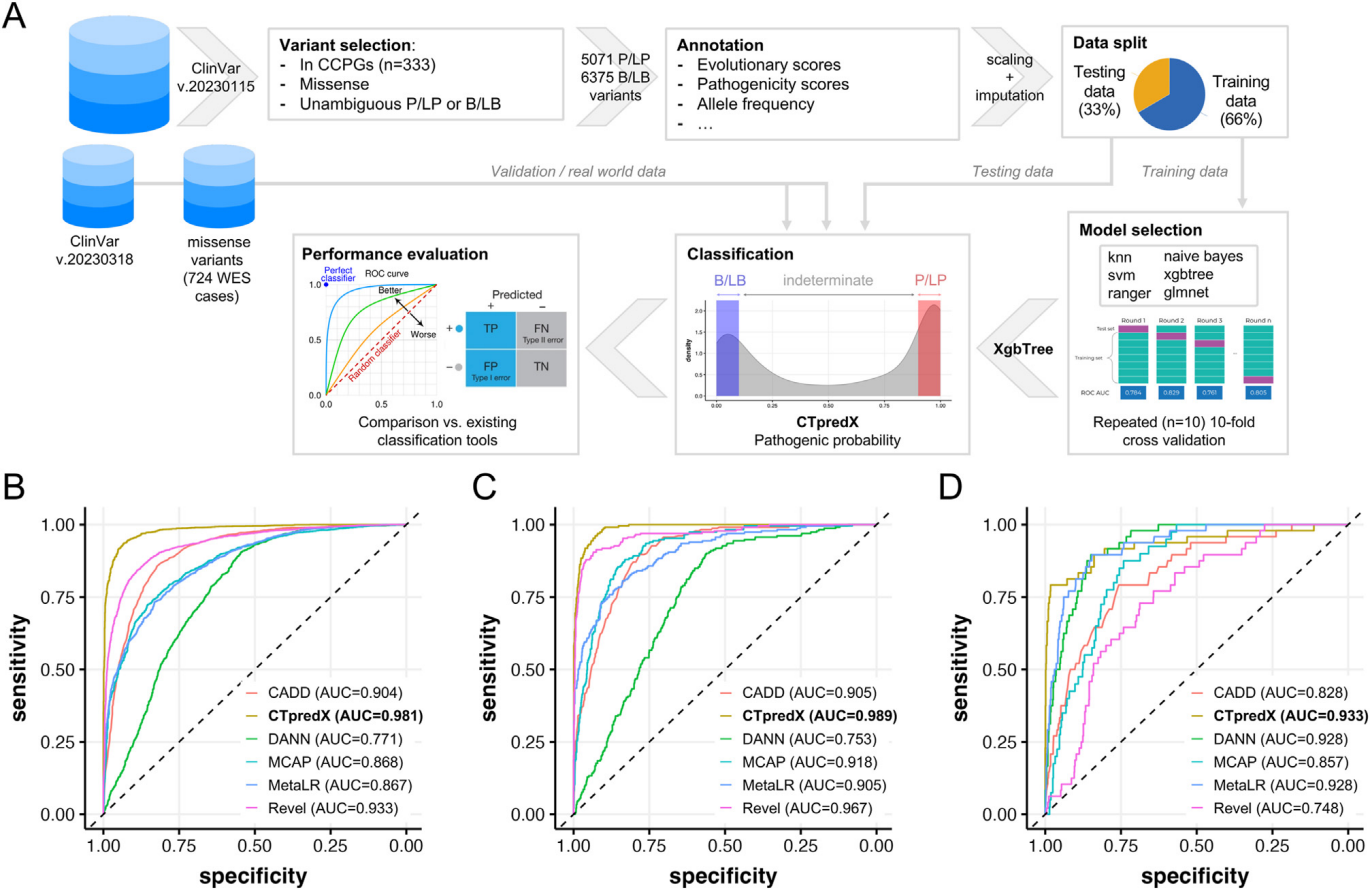
CTpredX workflow. **(A)** The ClinVar dataset (variants up to January 15, 2023) was filtered to only retain un-conflicting P/LP and B/LB missense variants in childhood cancer predisposing genes (CCPGs) and processed with annotation, scaling, and imputation of missing values (see supplementary methods). A data split with a ratio of 2:1 was used to define a training and a test dataset. The training set was used to optimize six machine learning algorithms and to select the best-performing one based on ROC-AUC in a repeated 10-fold cross-validation. CTpredX (developed on the best performing algorithm, XgbTree) was benchmarked against existing whole-genome prediction tools using the holdout testing dataset, against an additional independent test set (validation) built using variants annotated in ClinVar between January 15 and March 18, 2023, and against a set of missense variants identified in an internal cohort of 724 cases with neuroblastoma. At actionable levels, variants are classified into the following categories according to the pathogenicity probability: disease-causing (CTpredX score ≥ 0.9), benign/likely benign (CTpredX score ≤ 0.1), and a clinically indeterminate group of variants of uncertain significance with low interpretative confidence (0.1 < CTpredX score <0.9). **(B**–**D)** Receiver operating characteristic curves for CTpredX in conjunction with the five tested tools. Performance in predicting the pathogenicity of variants from the holdout testing dataset (B), from the independent validation dataset (C), and from the real-world dataset (D), including missense variants found in 724 whole-exome sequencing studies reported with automated ACMG/AMP classification. Different colors represent the six test scores. The gray line is the reference corresponding to the performance of a classifier that completely and randomly classifies the condition.

Variant annotation was performed using the Ensembl Variant Effect Predictor (VEP), and 34 functional annotations were used as features for six different machine learning classifiers (summary statistics reported in Table S2). The best-performing classifier was selected based on the area under the receiver operating characteristic curve (AUC-ROC) after repeated ten-fold cross-validation. A stepwise grid search was then employed for hyperparameter fine-tuning, resulting in the construction of the final predictive model, CTpredX. The tool's performance was compared against five broadly used whole-genome prediction scores, namely CADD, M-CAP, Revel, DANN, and META-LR, in terms of AUC-ROC. A 90% high-confidence classification threshold was then applied to classify variants into one of three categories: P/LP (CTpredX score ≥0.9), B/LB (CTpredX score ≤0.1), and indeterminate (0.1 < CTpredX score <0.9), and performance was also measured in terms of accuracy. For accuracy measurement of the existing prediction scores, we used the “supporting” *in silico* thresholds recommended by the ClinGen consortium for inclusion in the ACMG/AMP guidelines for PP3 and BP4 criteria (summarized in Table S3).^3^

Our model selection procedure identified Extreme Gradient Boosting (XgbTree) as the best-performing algorithm in terms of AUC-ROC (mean AUC-ROC = 0.981; Fig. S1). XgbTree was also the most stable (SD AUC-ROC = 0.18) across resamples compared with the other tested algorithms. To evaluate the CTpredX performance, we used the holdout testing dataset. CTpredX was compared against five well-established genome-wide prediction scores (CADD, M-CAP, Revel, DANN, and META-LR) reported to perform well in the impact prediction of missense variants.^4^

Classification performance was first summarized using the AUC-ROC and CTpredX achieved the best score (AUC-ROC = 0.975), followed by Revel (AUC-ROC = 0.906) and CADD (AUC-ROC = 0.886; Fig. 1B and Table S4). The difference in performance was statistically significant, with increased AUC-ROC (maximum *p*-value <0.01 from pairwise comparisons using DeLong's test; Table S5).

Additionally, to further evaluate classification performance at actionable levels, we assessed the accuracy at thresholds corresponding to “supporting” levels of certainty required for clinical decision-making (see definitions in Table S3) based on ClinGen recommendations for inclusion in PP3/BP4 ACMG criteria.^3^ Using these thresholds, CTpredX also outperformed existing variant classification tools in terms of accuracy when assessed with the holdout testing dataset. Overall, CTpredX assigned high-confidence classifications to 85% of test variants (3306 out of 3891) and maximized the identification of both P/LP and B/LB variants, yielding the highest accuracy (Fig. S2 and Table S4). In total, CTpredX correctly classified 97.5% of test variants with a confidence level of 90% or greater. The proportion of correctly classified variants was significantly higher (maximum *p*-value < 0.01 from pairwise comparisons) than those obtained with other tested tools. CTpredX only scored lower than Revel in terms of the percentage of indeterminate variants (15% *vs*. 13.9%; Table S4).

In line with previous results, CTpredX consistently achieved the highest score (AUC-ROC = 0.989) when compared with existing tools on an independent dataset that included variants non overlapping with training/testing datasets but unambiguously reported in ClinVar between January 15 and March 18, 2023 (Fig. 1C). Despite a slightly higher percentage of indeterminate variants (22.6%), it classified B/LB and P/LP variants with the highest accuracy using binary classification at over 90% certainty (Table S4).

More than 90% of ClinVar variants in CCPGs have conflicting pathogenicity annotations, therefore, we sought to re-analyze these variants using CTpredX and provide a reclassification based on our predictor. Of 125,489 variants of uncertain significance, CTpredX was able to reclassify 56%. Specifically, 37.8% were reclassified as B/LB and 18.3% as P/LP, while 43.9% remained of uncertain significance (Fig. S3). We compared this classification with that performed by the Automated Germline Variant Pathogenicity (AutoGVP) tool,^5^ a recently developed system that follows ACMG/AMP guidelines by integrating germline variant pathogenicity annotations from ClinVar and sequence variant classifications from a modified version of InterVar. Not surprisingly, AutoGVP reclassified fewer variants (*n* = 238; 0.2%) due to more stringent ACMG/AMP criteria. However, 79% of these variants (*n* = 188) were commonly reclassified by both tools, showing high concordance (99%), while only two variants (ClinVar ID: 690489 and 691157) were inversely classified (B/LB by CTpredX and P/LP by AutoGVP).

CTpredX was finally tested on an internal cohort of 724 germline whole-exome sequencing samples from neuroblastoma patients.^2^ Using AutoGVP's classifications as ground truth, CTpredX outperformed other computational tools, achieving the highest AUC-ROC (0.933; Fig. 1D) and superior accuracy at 90% confidence thresholds (Table S4).

Predicting the impact of missense mutations remains a significant challenge in human genetics, particularly because most amino acid substitutions are classified as variants of uncertain significance. Many existing tools predict the impact of missense variants, but few are designed for specific diseases or disease groups. CTpredX addresses this limitation by exploiting a tailored prediction model for a curated set of CCPGs. By leveraging ClinVar annotations and employing a machine learning approach on multiple predictors, CTpredX demonstrated superior performance in terms of AUC-ROC and accuracy in both testing and validation datasets.

Significantly, our model can also detect variants not yet annotated in ClinVar but showing features similar to those classified as pathogenic in the database, potentially identifying overlooked pathogenic variants.

Despite promising performance, CTpredX has some limitations. Currently, it only predicts the pathogenicity of missense variants. Other variant types, such as insertions/deletions or regulatory variants, may contribute to childhood cancer pathogenesis, but these were not included due to a lack of high-confidence data. CTpredX is specifically trained on CCPG variants, therefore, its predictions for other diseases may not be as accurate. Extending the model to other diseases would require a re-training on disease-specific variants. Finally, CTpredX is not intended as a standalone clinical decision-making tool but rather as an adjunct to established criteria, such as ACMG/AMP guidelines, for ambiguous cases. CTpredX is accessible through a user-friendly web interface at https://ctpredx.ceinge.unina.it, enabling broader use in clinical and research environments.

## Funding

This work was supported by grants from the Association of Paediatric Oncology and Neuroblastoma ONLUS Naples (grant name: Editor), Italian Association for the Fight against Neuroblastoma (grant name: AlterAction), Italian Association for Cancer Research (grant number: 25796), Ministry of Health (grant name: PRIN PNRR 2022 P2022NFCPM).

## CRediT authorship contribution statement

**Ferdinando Bonfiglio:** Writing – review & editing, Writing – original draft, Visualization, Methodology, Formal analysis, Data curation, Conceptualization. **Vito Alessandro Lasorsa:** Methodology, Data curation. **Giampiero Pirozzi:** Visualization. **Achille Iolascon:** Writing – review & editing, Writing – original draft, Supervision, Funding acquisition. **Mario Capasso:** Writing – review & editing, Writing – original draft, Supervision, Funding acquisition, Conceptualization.

## Conflict of interests

The authors confirm that there is no conflict of interests.
