# Supplementary material for: CTpredX: Enhancing missense variant pathogenicity prediction in childhood cancer predisposition genes
Source: Genes Dis. 2025 Apr 28;13(1):101661. doi: 10.1016/j.gendis.2025.101661 (PMC12466125; doi:10.1016/j.gendis.2025.101661)
Supplement: Multimedia component 1 [file mmc1.pdf]

SUPPLEMENTARY MATERIALS

CTpredX: Enhancing Missense Variant Pathogenicity Prediction in Childhood Cancer Predisposition Genes

SUPPLEMENTARY METHODS

Selection of childhood cancer genes

A total of 333 genes (**Table S1**) associated with childhood cancer were selected using an approach based on the integration of literature and database search. In particular, genes were selected through the harmonization of gene identifiers from the lists published in Fiala et al.,[1] Wagener et al.,[2] Wang et al.,[3] and integrated with the genes from "*Childhood solid tumours cancer susceptibility*" and "*Tumour predisposition childhoood onset*" from PanelApp, a portal of publicly available NGS gene panels (**Table S1**).[4]

Train, testing and validation dataset definition

To build the machine learning models, variants reported in ClinVar up to 2023-01-15 were exploited.[5] Only variants with no conflicting clinical interpretation were included. A total of 11,446 unique missense variants, reported to be pathogenic/likely pathogenic (P/LP, N=5,071) or benign/likely benign (B/LB, N=6,375) in established CCPGs were collected and subsequently split into training and testing datasets with a ratio of 2:1.

Two additional testing datasets (hereinafter referred to as validation datasets) were obtained. For the first one we used data reported in ClinVar between 2023-01-15 and 2023-03-18. This independent dataset consisted of 1,210 variants (233 P/LP and 977 B/LB) selected with the same criteria as described above. For the second one, we used missense variants found in a cohort of 724 neuroblastoma cases, already used in other studies[6] and selected P/LP and B/LP using the Automated Germline Variant Pathogenicity (AutoGVP),[7] a recently developed tool that integrates germline variant pathogenicity annotations from ClinVar and sequence variant classifications from a modified version of InterVar.[8]

Data preprocessing and model construction

Predicted molecular consequences and variant annotation for the whole dataset were obtained with Ensembl Variant Effect Predictor (version 104 for hg19/GRCh37 human genome assembly) on canonical transcripts. For each variant, annotated using the dbNSFP plugin,[9] we collected 34 functional annotations (**Table S2**) that were exploited as features for the machine learning classifier.

We conducted a z-score normalization of the training dataset, carried out by subtracting the mean of the feature from each value and then dividing by the standard deviation. Variants with more than 10 missing annotations were removed from the dataset and features were imputed using condition mean if missing. In order to avoid introducing biases in downstream modeling, features of testing and validation datasets were also standardized using the means and standard deviation of the training data; missing values in the testing and validation datasets were also imputed using the mean of each feature derived from the training data.

We selected six classification algorithms representative of the major families of machine learning algorithms (glmnet, k-Nearest Neighbors, naive bayes, Random Forest, Support Vector Machines, eXtreme Gradient Boosting), and applied a repeated (n=10) tenfold cross-validation to select the best algorithm using Caret R package.[10,11] In the repeated tenfold cross-validation of training dataset, the optimized candidate algorithms were compared among them and the best-performing one was selected according to a conventional threshold-independent performance measure, namely area under the receiver operating characteristic curve (ROC-AUC), a widely used metric for binary classification problems that describes the ability of the model to separate the classes (P/LP and B/LB), especially suited for imbalanced classes. After the model selection, a final predictive model (CTpredX) was constructed by training the best-performing algorithm on the whole set of training variants using a stepwise grid search for hyperparameters tuning. Variable importance score, a score that indicates how useful each feature was in the construction of the model the was calculated by Caret default functions.[10] A web application with a user-friendly interface for online query of variants of

interest was set-up with R-shiny (https://CRAN.R-project.org/package=shiny) and pre-computed CTpredX scores for all known missense variants in the selected CCPGs.

Benchmarking against existing whole-genome prediction tools

CTpredX predicts a pathogenicity score, ranging from 0 to 1, for each tested variant representing its estimated probability of being P/LP. The performance of the tool was compared against five broadly used whole-genome prediction scores, namely CADD,[12] M-CAP,[13] Revel,[14] DANN[15] and META-LR[16] in terms of ROC-AUC. Differences in ROC-AUC curves were tested with DeLong's test (*test.auc* function from the pROC R package).

In addition, to focus on performances at higher actionable thresholds, and compare pathogenic versus non-actionable indeterminate or benign/likely benign, we applied a 90% high-confidence classification threshold to classify variants into one of three categories: P/LP (CTpredX score ≥ 0.9), B/LB (CTpredX score ≤0.1) and indeterminate (0.1 < CTpredX score <0.9). We compared this classification with existing tools using the supporting *in silico* thresholds suggested by the ClinGen consortium for inclusion in the ACMG/AMP guidelines for PP3 and BP4 criteria (summarized in **Table S3**).[17] For this comparison, accuracy was used as primary performance metric. Accuracy takes into consideration both the sensitivity and specificity of the model and describes what proportion of all variants were correctly classified by that tool. Differences in accuracy were tested using Fisher's exact test and significance threshold was set at P=0.01.

SUPPLEMENTARY REFERENCES

1. Fiala EM, Jayakumaran G, Mauguen A, et al. Prospective pan-cancer germline testing using MSK-IMPACT informs clinical translation in 751 patients with pediatric solid tumors. *Nat Cancer*. 2021;2:357-365. doi:10.1038/s43018-021-00172-1
2. Wagener R, Taeubner J, Walter C, et al. Comprehensive germline-genomic and clinical profiling in 160 unselected children and adolescents with cancer. *Eur J Hum Genet*. 2021;29(8):1301-1311. doi:10.1038/s41431-021-00878-x
3. Wang Z, Wilson CL, Easton J, et al. Genetic Risk for Subsequent Neoplasms Among Long-Term Survivors of Childhood Cancer. *J Clin Oncol*. 2018;36(20):2078-2087. doi:10.1200/JCO.2018.77.8589
4. Martin AR, Williams E, Foulger RE, et al. PanelApp crowdsources expert knowledge to establish consensus diagnostic gene panels. *Nat Genet*. 2019;51(11):1560-1565. doi:10.1038/s41588-019-0528-2
5. Landrum MJ, Lee JM, Benson M, et al. ClinVar: improving access to variant interpretations and supporting evidence. *Nucleic Acids Research*. 2018;46(D1):D1062-D1067. doi:10.1093/nar/gkx1153
6. Bonfiglio F, Lasorsa VA, Aievola V, et al. Exploring the role of HLA variants in neuroblastoma susceptibility through whole exome sequencing. *HLA*. 2024;103(5):e15515. doi:10.1111/tan.15515
7. Kim J, Naqvi AS, Corbett RJ, et al. AutoGVP: a dockerized workflow integrating ClinVar and InterVar germline sequence variant classification. *Bioinformatics*. 2024;40(3):btae114. doi:10.1093/bioinformatics/btae114
8. Li Q, Wang K. InterVar: Clinical Interpretation of Genetic Variants by the 2015 ACMG-AMP Guidelines. *The American Journal of Human Genetics*. 2017;100(2):267-280. doi:10.1016/j.ajhg.2017.01.004
9. Liu X, Li C, Mou C, Dong Y, Tu Y. dbNSFP v4: a comprehensive database of transcript-specific functional predictions and annotations for human nonsynonymous and splice-site SNVs. *Genome Medicine*. 2020;12(1):103. doi:10.1186/s13073-020-00803-9
10. Kuhn M. Building Predictive Models in R Using the caret Package. *Journal of Statistical Software*. 2008;28:1-26. doi:10.18637/jss.v028.i05
11. R Core Team. R: A language and environment for statistical computing, Vienna, Austria. Published online 2013. http://www.R-project.org/
12. Rentzsch P, Witten D, Cooper GM, Shendure J, Kircher M. CADD: predicting the deleteriousness of variants throughout the human genome. *Nucleic Acids Research*. 2019;47(D1):D886-D894. doi:10.1093/nar/gky1016
13. Jagadeesh KA, Wenger AM, Berger MJ, et al. M-CAP eliminates a majority of variants of uncertain significance in clinical exomes at high sensitivity. *Nat Genet*. 2016;48(12):1581-1586. doi:10.1038/ng.3703
14. Ioannidis NM, Rothstein JH, Pejaver V, et al. REVEL: An Ensemble Method for Predicting the Pathogenicity of Rare Missense Variants. *Am J Hum Genet*. 2016;99(4):877-885. doi:10.1016/j.ajhg.2016.08.016
15. Quang D, Chen Y, Xie X. DANN: a deep learning approach for annotating the pathogenicity of genetic variants. *Bioinformatics*. 2015;31(5):761-763. doi:10.1093/bioinformatics/btu703
16. Dong C, Wei P, Jian X, et al. Comparison and integration of deleteriousness prediction methods for nonsynonymous SNVs in whole exome sequencing studies. *Human Molecular Genetics*. 2015;24(8):2125-2137. doi:10.1093/hmg/ddu733
17. Pejaver V, Byrne AB, Feng BJ, et al. Calibration of computational tools for missense variant pathogenicity classification and ClinGen recommendations for PP3/BP4 criteria. *The American Journal of Human Genetics*. 2022;109(12):2163-2177. doi:10.1016/j.ajhg.2022.10.013

SUPPLEMENTARY FIGURES

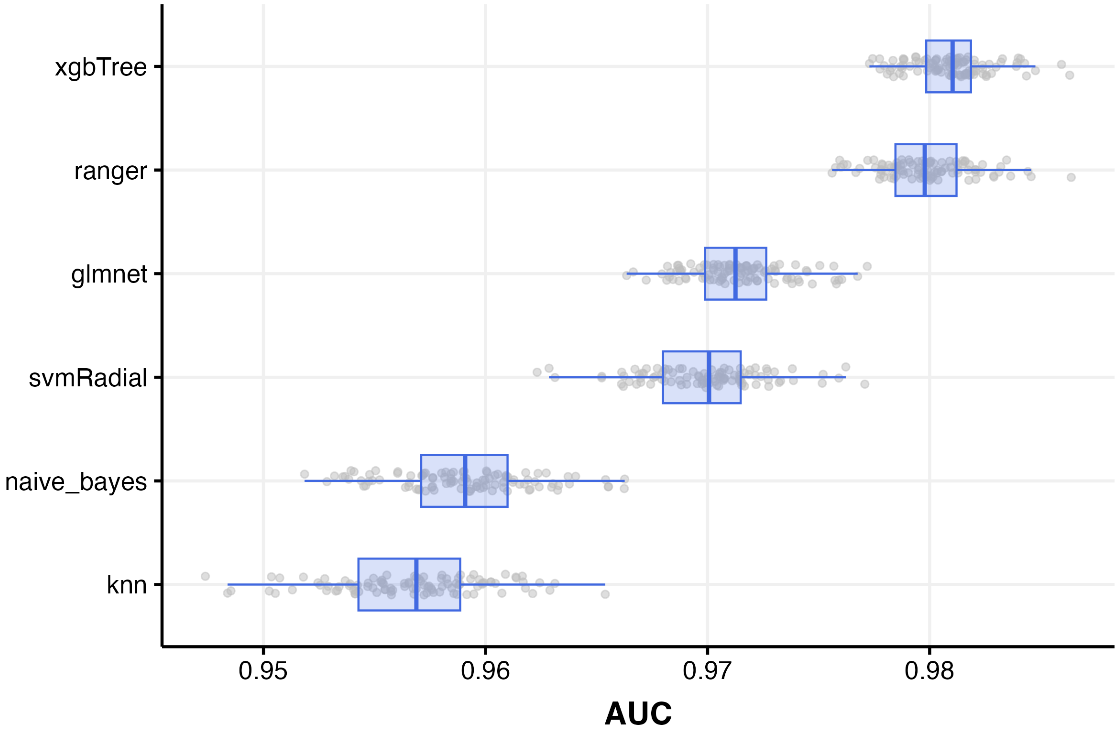

Figure S1. XgbTree algorithm performs best in the training set with repeated ten-fold cross validation. The plot shows summary statistics (mean and 95% confidence level) for the performance metric for the six tested models after repeated (n=10) ten-fold cross-validation for a total of 100 resamples (gray dots). The best performing model on resamples based on the mean AUC score was XgbTree.

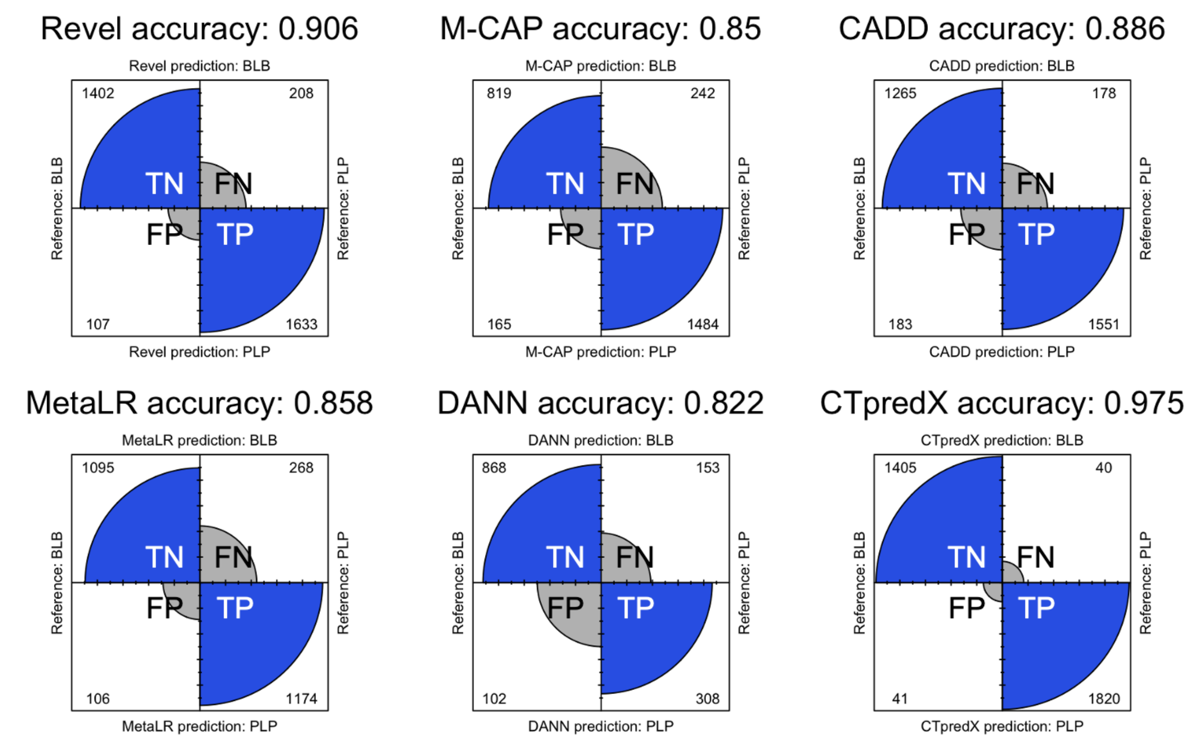

Figure S2. Accuracy comparison using the holdout testing dataset. Fourfold plot synopsizing the quantity of true positive (TP), true negative (TN), false positive (FP), and false negative (FN) for CTpredX predictions on the holdout testing data set in comparison with the 5 existing tools. Accuracy is reported on the top of each plot.

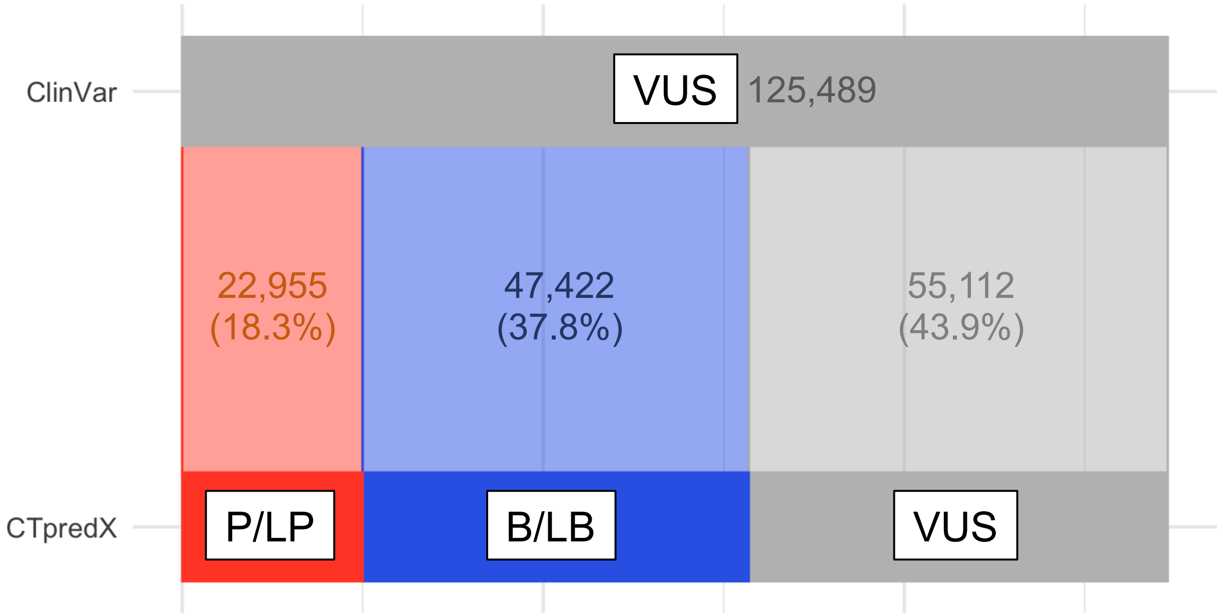

Figure S3. Proportion and numbers of ClinVar VUS variants reclassified by CTpredX.
